# Anti-Atrophic Effects of Dichotomine B from *Stellaria dichotoma* During Starvation-Induced Skeletal Muscle Atrophy

**DOI:** 10.3390/molecules30183839

**Published:** 2025-09-22

**Authors:** Jae-Yong Kim, Uttapol Permpoon, Ju-hee Lee, Ji Hoon Kim, Hye Mi Kim, Je-Seung Jeon, Tae-gyu Nam, Chul Young Kim

**Affiliations:** 1 College of Pharmacy and Institute of Pharmaceutical Science and Technology, Hanyang University ERICA, Ansan 15588, Gyeonggi-do, Republic of Korea; kjaey0331@huons-n.com (J.-Y.K.); uttapol.pem@gmail.com (U.P.); 0702leeeun@naver.com (J.-h.L.); gg890718@gmail.com (J.H.K.); hyemi586@hanyang.ac.kr (H.M.K.); 2 Lifecare Center, Huons N Co., Ltd., Gwacheon-si 13840, Gyeonggi-do, Republic of Korea; 3 Rural Development Administration (RDA), Eumseong-gun 27709, Chungcheongbuk-do, Republic of Korea; jsjeoncy@korea.kr

**Keywords:** *Stellaria dichotoma*, skeletal muscle atrophy, dichotomine B, starvation, dexamethasone

## Abstract

Muscle atrophy is defined as reductions in muscle size and function and represents a critical concern affecting elderly populations, immobilized patients, and individuals following specific dietary regimens, such as fasting and low-protein diets. This study investigated the protective effects of *Stellaria dichotoma* root extract and its isolated bioactive compounds during muscle atrophy using both in vitro and in vivo experimental models. First, *S. dichotoma* root extract prevented dexamethasone (DEX)-induced atrophy in C2C12 myotubes. Through systematic solvent partitioning and resin chromatography, five compounds (**1**–**5**) were successfully isolated from the *n*-butanol fraction. Dichotomine B (**2**) was identified as the most abundant and bioactive constituent. Treatment with dichotomine B significantly preserved the myotube diameter, enhanced the fusion index, and maintained the myosin heavy chain protein level while suppressing key atrophic biomarkers, including FoxO3a, MuRF-1, and Atrogin-1, in DEX-treated myotubes. Furthermore, dichotomine B (**2**) reduced proteolysis in serum-free cultured C2C12 myotubes and in mice subjected to 48 h of fasting, preserving muscle mass and strength. These findings suggest that *S. dichotoma* root extract and its principal compound, dichotomine B (**2**), have promising therapeutic potential and provide an opportunity to develop novel pharmacological interventions against muscle wasting through suppression of proteolysis pathways.

## 1. Introduction

Muscle atrophy, or muscle wasting, is defined as reductions in the size, mass, strength, and function of muscle fibers. It can be caused by several conditions [1]. Disused muscles; starvation; and chronic diseases such as hormone dysregulation, sepsis, inflammation, and cancer induce a typical muscle loss named cachexia [2,3]. The aging-related muscle atrophy called sarcopenia is currently increasing in prevalence in the modern world, corresponding to growth in the elderly population [4]. Muscle atrophy impacts overall quality of life, increases the risk of bone fractures, and correlates with cardiovascular diseases that shorten patients’ lifespans [5,6]. In addition, muscle atrophy not only affects physical activity but also impacts systemic metabolism, as muscle tissue serves a large storage reservoir for energy [7]. Exercise, rehabilitation, and nutritional support are generally recommended to prevent muscle loss; however, pharmacological therapy is still not available [8,9].

The balance of protein synthesis and degradation plays a central role in muscle biology [10]. Sufficient protein production maintains myotube thickness and function. In terms of molecular mechanisms, growth factor signals such as insulin-like growth factor 1 (IGF-1) and its receptor (IGF-1R) trigger mTOR complex 1 (mTORC1)-mediated protein synthesis through tyrosine phosphorylation and PI3K/Akt activation [11,12]. Some amino acids, like branched-chain amino acids (BCAAs: leucine, isoleucine, and valine) directly activate mTORC1 and support muscle function [13]. Therefore, starvation or a low-protein diet impairs protein synthesis and muscle strength [14]. Synthetic glucocorticoids (GCs), such as dexamethasone (DEX), inhibit growth receptor signaling by blocking phosphorylation of these receptors and suppress mTOR activation by upregulating mTORC1 suppressors, including regulated in development and DNA damage 1 (REDD1) and Kruppel-like factor 15 (KLF-15) [15,16].

Muscle protein catabolism is primarily driven by the ubiquitin–proteasome system (UPS), a complex cascade initiated by conjugation of ubiquitin to the target protein, followed by recruitment of several subunits to form a proteasome [17]. Muscle atrophy F-box (MAFbx or Atrogin-1) and muscle ring-finger protein 1 (MuRF-1) are muscle-specific ubiquitin E3-ligases [18]. Upregulation of Atrogin-1 and MuRF-1 negatively correlates with the protein levels of myosin heavy chain (MHC) and the myofiber diameter [19]. DEX treatment enhances Atrogin-1 and MuRF-1 expression, which induces muscle atrophy in vitro and in vivo [20]. DEX activates FoxO1 and FoxO3, which initiate transcription of several atrogenes, resulting in protein degradation and myotube size reduction [21,22]. In addition, the PI3K/Akt/mTOR axis interferes with the UPS by inhibiting FoxO3 nuclear translocation in muscle tissue [23,24]. Lacking a growth signal decreases protein synthesis and increases protein degradation. Thus, suppression of Atrogin-1 and MuRF-1 expression prevents muscle wasting in various conditions.

In the modern world, the importance of traditional medicine is reduced as it is replaced by chemically synthesized drugs; however, traditional medicine still guides scientists to discover new natural products and their derivatives. This led to drug discovery and the development of several natural product-derived compounds that were recently tested in clinical trials [25]. The dried root of *Stellaria dichotoma* L. var. *lanceolata* Bge. has been used in traditional Asian medicine for several decades and has recently been studied in modern phytotherapy [26]. *S. dichotoma* roots have broad benefits in various hyperthermal conditions, inflammation-related disorders, childhood malnutrition, obesity, febrile diseases, insomnia, and aging [27,28]. Among several compounds isolated from *S. dichotoma* roots, a group of β-carboline indole alkaloids named dichotomines were identified as the most abundant. They exhibit immunosuppressive activity by reducing NO production in LPS-stimulated RAW 264.7 cells [29]. A tricyclic pyrido [3,4-b] indole ring is preserved as a core structure in all derivatives of β-carboline alkaloids, which are found in several natural products. β-carboline alkaloids demonstrate several benefits in broad diseases such as cancers, tuberculosis, malaria, and Alzheimer’s disease [30,31].

However, the effects of *S. dichotoma* roots and their isolated compounds on muscle tissue have not been clarified. In this article, we demonstrate that the extract of *S. dichotoma* roots and its isolated compounds provide protective effects against muscle atrophy induced by DEX treatment and starvation. Furthermore, we found that dichotomine B, a major active constituent, showed promising results in both in vitro and in vivo models of starvation-induced muscle atrophy.

## 2. Results

### 2.1. Bioactivity-Guided Isolation of Compounds ***1**–**5*** Using Dexamethasone-Induced C2C12 Myotube Atrophy Model

**Figure 1:**
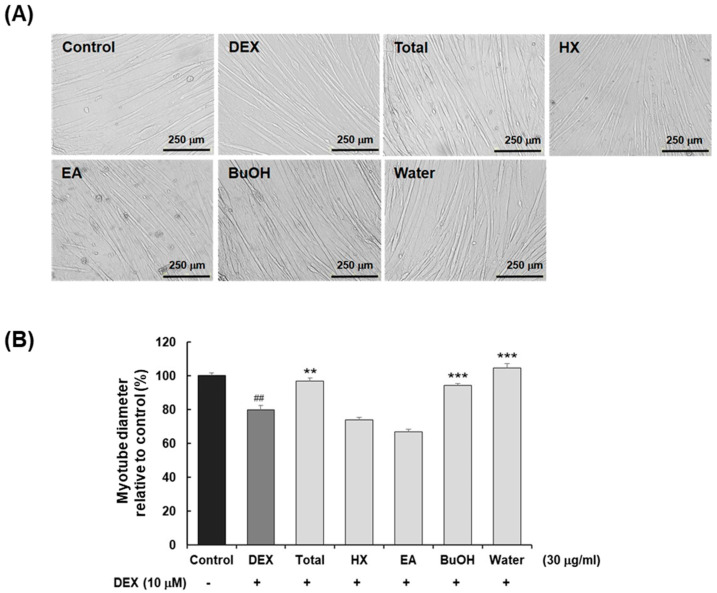
The effects of *S. dichotoma* extract and its sub-fractions on the diameter of DEX-induced C2C12 myotubes. (**A**) Cell morphology and (**B**) relative changes in the diameter of DEX-induced C2C12 myotubes cotreated with each *S. dichotoma* extract (30 μg/mL) observed under a microscope (200×) and analyzed by ImageJ2 software. The data are presented as means ± SDs (n = 3). ^##^ *p* < 0.01 vs. control. ** *p* < 0.01 vs. dexamethasone treatment. *** *p* < 0.001 vs. dexamethasone treatment.

Treatment with the crude ethanolic extract (30 μg/mL) significantly attenuated DEX-induced myotube atrophy. Specifically, DEX treatment alone resulted in a 20.1% reduction in the myotube diameter compared to the untreated control, whereas cotreatment with the *S. dichotoma* extract led to a 21.4% increase in diameter relative to the DEX-only group (Figure 1).

Following solvent partitioning, each fraction was assessed for bioactivity. Both the *n*-butanol and water fractions exhibited preventive effects against muscle atrophy. Based on HPLC analysis, the *n*-butanol extract was selected for further investigation to isolate bioactive constituents (Figure S1). Five major peaks were found in the *n*-butanol fraction, which were subsequently separated using Diaion HP-20 macroporous resin chromatography, followed by prep-HPLC (Figures S2 and S3).

**Figure 2:**
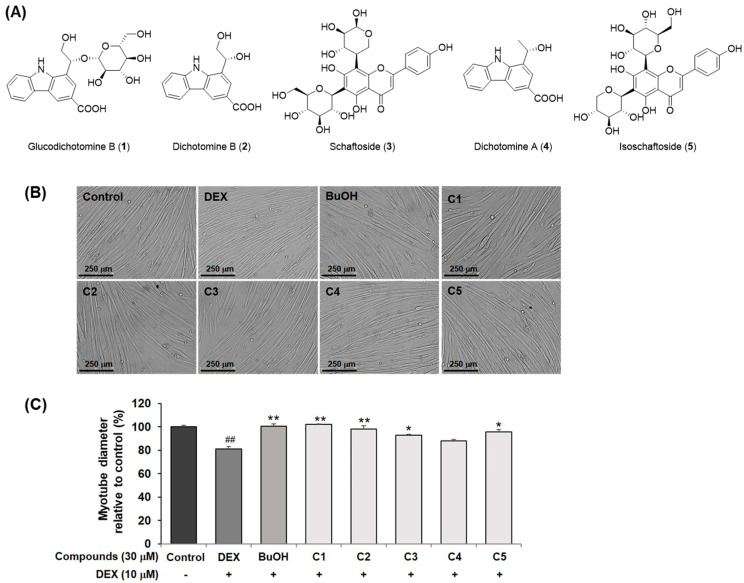
The effects of isolated compounds **1**–**5** from *S. dichotoma* on the diameter of DEX-induced C2C12 myotubes. (**A**) The chemical structures of compounds **1**–**5** isolated from the *n*-butanol fraction. (**B**) The cell morphology and (**C**) relative changes in myotube diameter of DEX-induced C2C12 myotubes cotreated with each isolated compound (**1**–**5**) under a microscope (scale bar = 250 μm) and analyzed by ImageJ software. These results are presented as means ± SDs (n = 3). ^##^ *p* < 0.01 vs. control. * *p* < 0.05 vs. dexamethasone treatment. ** *p* < 0.01 vs. dexamethasone treatment.

The chemical structures of the isolated compounds (**1**–**5**) were elucidated as glucodichotomine B (**1**), dichotomine B (**2**), shaftoside (**3**), dichotomine A (**4**), and isoshaftoside (**5**) (Figure 2A and Figures S4–S8). Next, the bioactivities of isolated compounds **1**–**5** were evaluated in C2C12 myotubes. None of the compounds exhibited cytotoxicity at a concentration of 30 μM (Figure S9). The *n*-butanol extract and compounds **1**–**3** and **5** significantly preserved the cell diameter in DEX-cotreated C2C12 myotubes compared to DEX treatment alone (Figure 2B,C). These results indicate that *S. dichotoma* extract and active compounds **1**–**3** and **5** potentially prevent DEX-induced myotube atrophy.

Furthermore, dichotomine B (**2**) was identified as the most abundant compound in the ethanol extract, which correlated with anti-atrophic activity observed across various ethanol–water composition ratios (Figure S10). Therefore, considering the amounts and activities of isolated compounds **1**–**5**, dichotomine B (**2**) was selected for further studies.

### 2.2. Dichotomine B Ameliorates Dexamethasone-Induced Skeletal Muscle Atrophy by Inhibiting Protein Degradation

**Figure 3:**
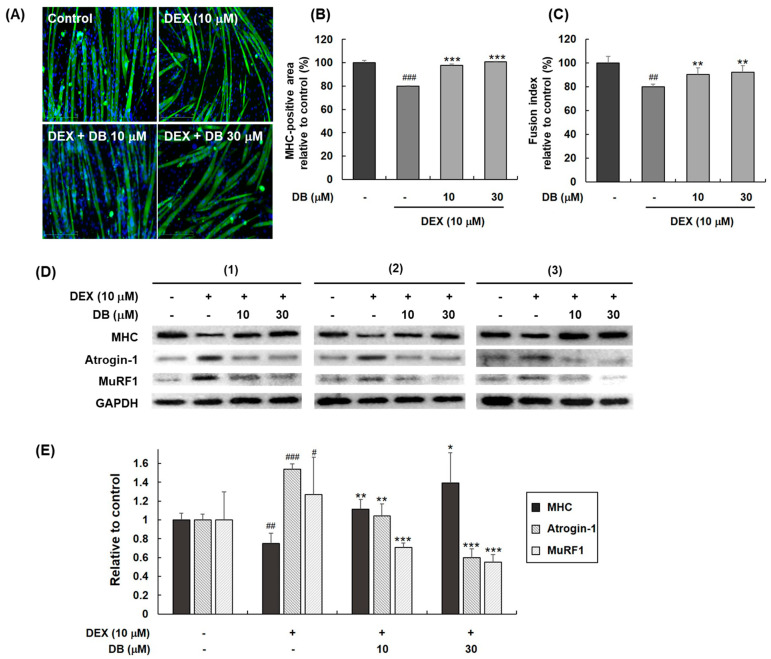
The effects of dichotomine B during DEX-induced C2C12 myotube atrophy. (**A**) Immunofluorescence staining of MHC after cotreatment with DEX and dichotomine B (DB) (10 or 30 μM) in C2C12 myotubes for 24 h. (**B**) The MHC-positive area and (**C**) fusion index were analyzed and determined by ImageJ2 software. The effects of DB on muscle atrophy biomarkers in DEX-stimulated C2C12 myotubes. (**D**) Western blot analysis of MHC, Atrogin-1, and MuRF-1 from lysates of DEX-induced C2C12 myotubes cotreated with DB (10 or 30 μM) for 24 h. The experiment was performed in triplicate independently (from the left panel to the right panel). (**E**) The relative changes in the protein levels of MHC, Atrogin-1, and MuRF-1 in DB-treated myotubes. The experiments were performed in triplicate. The data are presented as means ± SDs (n = 3). # *p* < 0.05 vs. control. ## *p* < 0.01 vs. control. ### *p* < 0.001 vs. control. * *p* < 0.05 vs. dexamethasone treatment. ** *p* < 0.01 vs. dexamethasone treatment. *** *p* < 0.001 vs. dexamethasone treatment.

To evaluate the protective effects of dichotomine B (**2**) during DEX-induced myotube atrophy, an immunofluorescence assay was performed to detect MHC expression. Treatment with DEX (10 μM) for 24 h resulted in a marked reduction in C2C12 myotube thickness. However, cotreatment with dichotomine B at 10 or 30 μM effectively reversed this effect. The cotreatment groups showed significant increases in both the myotube diameter and the number of multinucleated myotubes (the fusion index) compared to the DEX-only group (Figure 3A–C).

Furthermore, we determined muscle atrophy markers using Western blot analysis. After 24 h of incubation, DEX-treated C2C12 myotubes exhibited a significant decrease in their MHC level, which correlated with the upregulation of Atrogin-1 and MuRF-1, key regulators of muscle protein degradation via the ubiquitin–proteasome system (Figure 3D,E). In contrast, dichotomine B counteracted these responses by increasing MHC levels and suppressing both Atrogin-1 and MuRF-1. These results indicate that dichotomine B from *S. dichotoma* effectively mitigates DEX-induced myotube atrophy.

### 2.3. Dichotomine B Maintains Myotube Thickness in Serum-Free in Vitro Conditions

**Figure 4:**
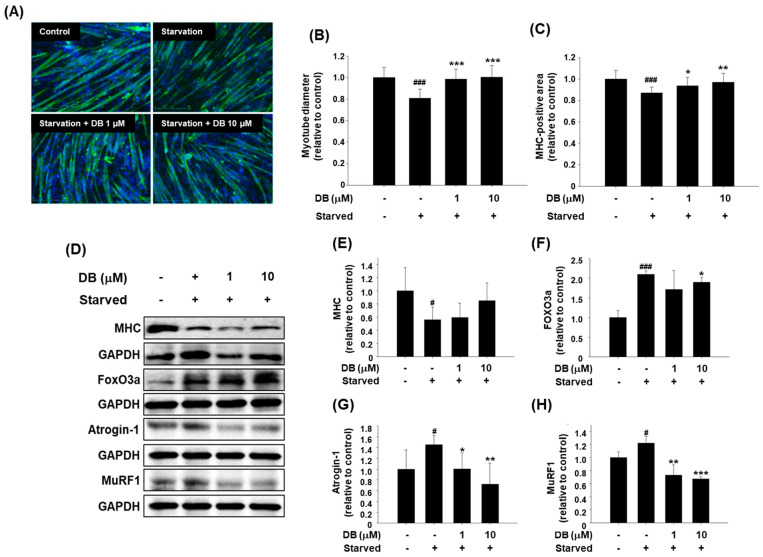
The effects of dichotomine B on the myotube diameter and MHC expression in C2C12 myotubes under serum-free conditions. (**A**) Immunofluorescence staining of MHC after treatment with dichotomine B (DB) (1 or 10 μM) in C2C12 myotubes under serum-free conditions for 24 h. (**B**) The myotube diameter and (**C**) the fusion index were analyzed and determined by ImageJ2 software. (**D**) Western blot analysis of MHC, Atrogin-1, and MuRF-1 from lysates of C2C12 myotubes incubated with DB (1 or 10 μM) under starvation conditions for 24 h. The relative changes in the protein levels of (**E**) MHC, (**F**) FoxO3a, (**G**) Atrogin-1, and (**H**) MuRF-1 in DB-treated myotubes. The data are presented as means ± SDs (n = 3). # *p* < 0.05 vs. control. ### *p* < 0.001 vs. control. * *p* < 0.05 vs. starved myotubes. ** *p* < 0.01 vs. starved myotubes. *** *p* < 0.001 vs. starved myotubes.

To investigate whether dichotomine B prevents muscle protein degradation and the reduction in myotube thickness under nutrient-deprived conditions, the differentiation medium (2% HS-DMEM) was replaced with serum-free DMEM in the presence or absence of dichotomine B after myotube formation was completed. Dichotomine B did not affect the viability of C2C12 myotubes cultured in 2% HS-DMEM, but 10 μM dichotomine B significantly enhanced myotube viability under starvation conditions (Figure S11). Immunofluorescence staining of intracellular MHC revealed that dichotomine B preserved myotube thickness (Figure 4A). In addition, treatment with dichotomine B at 1 or 10 μM significantly increased both the myotube diameter and the MHC-positive area compared with starved myotubes (Figure 4B,C). Furthermore, the expression levels of atrophic markers, including FoxO3a, Atrogin-1, and MuRF-1, were reduced in a dose-dependent manner (Figure 4D,F–H). By contrast, the expression level of MHC tended to increase in starved myotubes treated with 10 μM dichotomine B, although the difference was not statistically significant (Figure 4D,E).

### 2.4. Dichotomine B Preserves Muscle Mass and Function in Starved Mice

**Figure 5:**
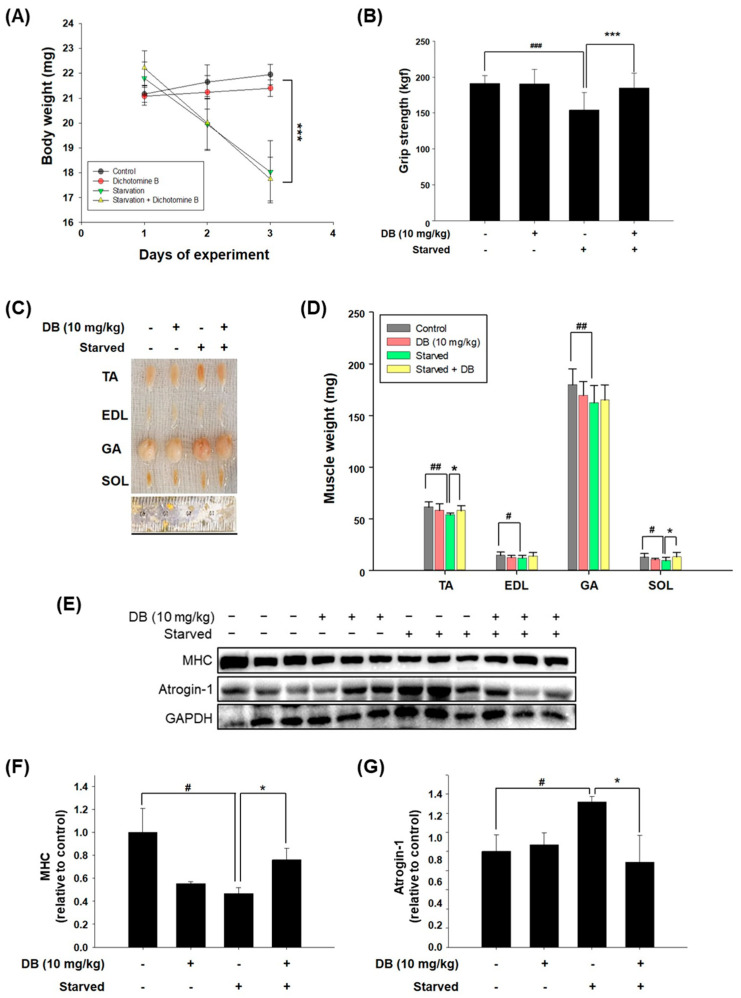
The effects of dichotomine B on grip strength, muscle weight, and atrophy biomarkers in fasting C57BL/6J mice. (**A**) The body weights of mice fed controlled standard chow or fasting with or without administration of dichotomine B (DB) at 10 mg/kg and followed for up to 2 days. (**B**) The grip strength test was performed on day 3 of the experiment. (**C**,**D**) The tibialis anterior (TA), extensor digitorum longus (EDL), gastrocnemius (GA), and soleus (SOL) were collected on day 3 of the experiment to measure their diameters and weights. (**E**) Western blot analysis of MHC and Atrogin-1 from mouse TA muscles. The relative changes in the protein levels of (**F**) MHC and (**G**) Atrogin-1 in each group. The data are presented as means ± SDs (n = 3). # *p* < 0.05 vs. the control group. ## *p* < 0.01 vs. the control group. ### *p* < 0.001 vs. the control group. * *p* < 0.05 vs. the starved group. *** *p* < 0.001 vs. the starved group.

To evaluate the effects of dichotomine B on muscle mass, we employed a starvation model in C57BL/6J mice. A single intraperitoneal injection of dichotomine B (10 mg/kg) was administered on the first day of fasting. Mice subjected to fasting began to lose body weight after 24 h, with a significant reduction observed at 48 h (Figure 5A). Notably, dichotomine B administration did not affect body weight in either control-fed or fasted mice.

Dichotomine B significantly preserved grip strength in the starved mice but conferred no additional benefit to the control-fed group (Figure 5B). Moreover, dichotomine B treatment prevented the loss of muscle mass in the starved mice, particularly in the tibialis anterior and soleus muscles (Figure 5C,D). Western blot analysis of tibialis anterior muscle lysates further demonstrated that dichotomine B significantly maintained MHC protein levels and suppressed Atrogin-1 expression under starvation conditions (Figure 5E–G). These in vivo results suggest that dichotomine B effectively prevents muscle wasting and preserves muscle function during nutrient deprivation, although it does not induce muscle hypertrophy under normal dietary conditions.

## 3. Discussion

In this study, the protective effects of *S. dichotoma* root extract and its isolated compounds (**1**–**5**) were investigated in C2C12 myotubes, and dichotomine B, the most abundant compound, was shown to prevent myotube atrophy. Dichotomine B has also been studied in various disease models. For example, it has been reported to inhibit pro-inflammatory cytokine secretion in lipopolysaccharide- and adenosine triphosphate (ATP)-stimulated microglia [32]. Additionally, dichotomine B is a key bioactive compound that mitigates Alzheimer’s disease pathology by inducing autophagy and activating the PI3K/Akt/mTOR and AMPK signaling pathways [33].

DEX treatment reduced the myotube diameter, decreased cell viability, and lowered MHC protein levels while increasing the expression of atrophy-related markers, including FoxO3a, MuRF-1, and Atrogin-1 [34]. MHC, a key component of myosin, plays an important role in muscle development, formation, and contraction [35]. Here, we used immunofluorescence staining and Western blot analysis to observe the myotube cell diameter and determine the expression of protein biomarkers, respectively. The crude EtOH extract from *S. dichotoma* exerted a protective effect in DEX-stimulated C2C12 myotubes in the combination treatment. Next, we used multiple bilayer solvent partitions to separate these compounds. The crude *n*-BuOH extract, which contained five compounds, maintained the ability to inhibit C2C12 myotube atrophy induced by DEX treatment. After we isolated five compounds from the *n*-BuOH fraction using Diaion HP-20 chromatography, dichotomine B (compound **2**) was identified and suggested as a key active compound. Then, we demonstrated that 10 or 30 μM dichotomine B maintained MHC protein levels and suppressed the upregulation of MuRF-1 and Atrogin-1 in DEX-stimulated C2C12 myotubes. Furthermore, dichotomine B preserved the cell diameter and significantly increased the fusion index compared with DEX treatment. A high fusion index indicates efficient myogenic differentiation, suggesting healthy myotube fiber formation [36]. Collectively, these results suggest that dichotomine B enhances myotube differentiation and prevents DEX-induced muscle atrophy.

Starvation is also a common cause of muscle wasting in humans, as it decreases protein synthesis and increases protein breakdown [37,38]. To prevent muscle atrophy, resistance training and supplementation of essential amino acids are recommended [39,40]. In terms of pharmacological therapy, some natural compounds and synthetic agents have been evaluated in pre-clinical and clinical studies, for instance, beta-hydroxy-beta-methylbutyrate and ursolic acid [41,42]. In our study, dichotomine B was also found to support muscle health during starvation. Dichotomine B at 1 or 10 μM maintained the myotube diameter and MHC protein levels in serum-free C2C12 myotubes for 24 h while suppressing the expression of FoxO3a, Atrogin-1, and MuRF-1. In an in vivo starvation model, dichotomine B did not affect body weight, but 10 mg/kg significantly preserved muscle strength during the grip test, correlating with maintained muscle mass. Additionally, dichotomine B prevented MHC reduction and Atrogin-1 induction in tibialis anterior tissue. These results suggest that dichotomine B may help maintain muscle mass and strength under conditions of food deprivation.

The role of dichotomine B with respect to muscle growth and prevention of muscle wasting is still unclear. Further, we plan to identify possible targets of dichotomine B in muscle cells. We also plan to chemically synthesize dichotomine B and its derivatives to study the impact of each functional group and increase the chance of improving their biological effects based on structure–activity relationship analysis.

## 4. Materials and Methods

### 4.1. Plant Material

*S. dichotoma* roots were purchased from Kyeongdong Oriental Herbal Market, Seoul, Republic of Korea, in October 2021 and identified by one of the authors (Chul Young Kim). A voucher specimen (HYUP-SD-001) was deposited in the Herbarium of the College of Pharmacy, Hanyang University.

### 4.2. Extraction and Isolation of Compounds ***1**–**5***

The *S. dichotoma* roots (3.0 kg) were ground and extracted by reflux with 5 L of ethanol (three times for 3 h each), and the solvents were evaporated in vacuo at 40 °C, yielding the ethanol extract (366.3 g). The crude extract (120 g) was suspended in 1 L of water and successively partitioned with the same volume of *n*-hexane, ethyl acetate, and *n*-butanol to give *n*-hexane (2.62 g), ethyl acetate (3.96 g), *n*-butanol (6.44 g), and water (103.53 g) extracts. The crude *n*-butanol extract (6 g) was subjected to Diaion HP-20 macroporous resin (Mitsubishi Chemical Co., Tokyo, Japan) chromatography, eluted with gradient mixtures of methanol and water (0:100 to 100:0, *v*/*v*), and washed with acetone to give 14 fractions (F1−F14).

The F-8 fraction (600 mg, 35% methanol eluent) was subjected to semi-preparative HPLC using an RP column (Cosmosil 5C18-MS-II, 250 × 20 mm I.D., Nacalai tesque, Kyoto, Japan) with gradient elution of acetonitrile and water with 0.1% trifluoroacetic acid (0–50 min, 5–20% acetonitrile) to yield compounds **1**–**5** (10 mg, 14.7 mg, 11.9 mg, 12.5 mg, and 4.6 mg, respectively). The detection wavelength was 265 nm, and the flow rate was 10 mL/min. The retention times of compounds **1**–**5** were 23.9 min, 23.1 min, 30.9 min, 33.6 min, and 34.0 min, respectively. A detailed isolation scheme is described in Supplementary Figure S2, and detailed spectral data of isolated compounds **1**–**5** are presented in Supplementary Figures S4–S8.

### 4.3. C2C12 Cell Culture and Differentiation

A mouse myoblast C2C12 cell line was purchased from the American Type Culture Collection (CRL-1772, ATCC) (Manassas, VA, USA) and cultured in Dulbecco’s modified Eagle medium (DMEM) high glucose supplemented with 10% heat-inactivated fetal bovine serum (FBS) and 1% penicillin/streptomycin at 37 °C with 5% CO_2_. For myotube differentiation, C2C12 myoblasts were seeded onto plates with a density of 6 × 10^4^ cells per mL of culture medium. When the cells reached 80–90% confluence, they were washed once with PBS and cultured with a differentiation medium, DMEM containing 2% horse serum (HS). Fresh differentiation medium was added every other day for up to 6 days.

### 4.4. Myotube Treatments and Starvation

After differentiation, the C2C12 myotubes were treated with 10 μM DEX (D4902, Sigma-Aldrich, Saint Louis, MO, USA) in the presence or absence of *S. dichotoma* extracts (30 μg/mL), isolated compounds (30 μM), or dichotomine B (10 or 30 μM) for 24 h. A differentiation medium containing 0.1% DMSO was used as a control. For the starvation assays, the differentiation medium was replaced with a serum-free medium with or without dichotomine B (1 or 10 μM), and incubation continued for 24 h. The myotube morphology was observed under a bright-field microscope.

### 4.5. Cell Viability Test

C2C12 myotube viability was measured by cell counting kit-8 (CCK-8) (Dojindo, CK04, Kumamoto, Japan). The old medium was discarded and replaced with a fresh medium containing 10% CCK-8 reagent. After incubation at 37 °C for 1–4 h, the absorbance at 450 nm was measured with a microplate reader. Sample turbidity was subtracted based on absorbance at 650 nm. The viability (%) was calculated compared to the control as follows:

Viability (%) = (O.D. test − blank/O.D. control − blank) × 100%(1)

### 4.6. Western Blot Analysis

Cells or mouse muscle tissues were lysed with an RIPA buffer (89900, Thermo Fisher Scientific, Waltham, MA, USA) containing a protease inhibitor cocktail at 4 °C for 30 min. The lysates were collected, and the protein concentration was measured with a BCA assay (23225 and 23227, Thermo Fisher Scientific). The protein was separated by 10% SDS–polyacrylamide gel electrophoresis and transferred to a PVDF membrane. After blocking with 3% BSA for 1 h, the membrane was incubated with a primary antibody (a mouse anti-MHC monoclonal antibody (clone B-5, sc-376157, Santa Cruz Biotechnology, Dallas, TX, USA), a rabbit anti-FoxO3a monoclonal antibody (clone 75D8, 2497, Cell Signaling Technology, Danvers, MA, USA), a mouse anti-MAFbx (Atrogin-1) monoclonal antibody (clone F-9, sc-166806, Santa Cruz Biotechnology), a mouse anti-MuRF-1 monoclonal antibody (clone C-11, sc-398608, Santa Cruz Biotechnology), or a mouse anti-GAPDH monoclonal antibody (clone H-12, sc-166574, Santa Cruz Biotechnology) at 4 °C overnight. The membrane was washed with TBS-T (0.05% Tween-20) and incubated with horseradish peroxidase-conjugated anti-mouse IgG (sc-516102, Santa Cruz Biotechnology) or horseradish peroxidase-conjugated anti-rabbit IgG (7074, Cell Signaling Technology) at RT for 1 h. After washing, a substrate was added, and the chemiluminescence signal was detected. The band intensity was determined by ImageJ2 software.

### 4.7. Myosin Heavy Chain Immunofluorescence Staining

Cells were washed with PBS twice and fixed with 4% paraformaldehyde at RT for 15 min. The cells were permeabilized with 0.1% Triton X-100 at 37 °C for 15 min. After blocking with 1% BSA for 1 h, a mouse anti-MHC monoclonal antibody was added, and the cells were incubated at 4 °C overnight. Then, the cells were washed with PBS and incubated with an Alexa Fluor 488-conjugated goat anti-mouse IgG monoclonal antibody (A-11001, Thermo Fisher Scientific) at RT for 1 h. Hoechst 33342 (H3570, Thermo Fisher Scientific) was used for nuclear staining. The stained cells were detected using fluorescence microscopy. The myotube diameter, MHC-positive area, and fusion index were analyzed by ImageJ2 software.

Fusion index = % of nuclei in MHC positive myotube/total nuclei in the field(2)

### 4.8. In Vivo Starvation Model

All animal procedures were approved by the Institutional Animal Care and Use Committee of Hanyang University (# HY-IACUC-23-0015), and this study was performed according to the guidelines of the National Institutes of Health. Male C57BL/6 mice (6 weeks old) were purchased from Koatech Laboratory Animal Company (Pyeongtaek, Republic of Korea) and maintained in the animal facility at the Hanyang University ERICA, Republic of Korea. All animals were housed under environmentally controlled standard conditions at 22 ± 2 °C with a relative humidity of 50–60% and maintained under a 12 h light/dark cycle. The mice were separated into four groups (*n* = 8 per group): (1) control (standard chow ad libitum), (2) dichotomine B (fed with dichotomine B administration), (3) starvation (48 h of fasting), and (4) starvation + dichotomine B (48 h of fasting with dichotomine B administration). Dichotomine B was administered via intraperitoneal injection with a dose of approximately 10 mg/kg on the first day of the experiment. For the starvation cohorts, food was removed for up to 48 h. Body weight was monitored every 24 h. After 48 h, a grip strength test was performed. Then, the mice were euthanized by exposure to CO_2_. The tibialis anterior, extensor digitorum longus, gastrocnemius, and soleus muscles were collected, their weights were measured, and lysates were prepared for Western blot analysis.

### 4.9. Statistical Analysis

All data are presented as means ± standard deviations (SDs) for at least three independent experiments. One-way analysis of variance (ANOVA), followed by Tukey’s post hoc test, was used to evaluate statistical significance using GraphPad Prism 5.04 (GraphPad Software Inc., La Jolla, CA, USA). A *p*-value lower than 0.05 was considered statistically significant.

## 5. Conclusions

These findings suggest that the bioactive compounds isolated from *S. dichotoma* root extract, especially dichotomine B, may have therapeutic potential for preventing muscle wasting during DEX treatment and starvation.

## Data Availability

Data will be made available upon request.

## Supplementary Materials

The following supporting information can be downloaded at https://www.mdpi.com/article/10.3390/molecules30183839/s1, Figure S1: Solvent fractionation of *S. dichotoma* extract and HPLC analysis; Figure S2: Isolation procedures using Diaion HP-20 chromatography; Figure S3: HPLC chromatograms of *n*-butanol extract and isolated compounds **1**–**5**; Figure S4: ^1^H and ^13^C NMR spectra of glucodichotomine B (**1**); Figure S5: ^1^H and ^13^C NMR spectra of dichotomine B (**2**); Figure S6: ^1^H and ^13^C NMR spectra of shaftoside (**3**); Figure S7: ^1^H and ^13^C NMR spectra of dichotomine A (**4**); Figure S8: ^1^H and ^13^C NMR spectra of isoshaftoside (**5**); Figure S9: Cell viability of compounds **1**–**5** (30 µM) in C2C12 myotubes after incubation for 24 h; Figure S10: Optimization of extraction solvents with various ethanol–water compositions for anti-atrophic activity during DEX-induced C2C12 myotube atrophy; Figure S11: Cell viability of dichotomine B (**2**) in C2C12 myotubes after incubation for 24 h; Table S1: Regression equations for isolated compounds **1**–**5**. References [43,44] are cited in the Supplementary Materials.
