# Supplementary material for: Anti-Atrophic Effects of Dichotomine B from Stellaria dichotoma During Starvation-Induced Skeletal Muscle Atrophy
Source: Molecules. 2025 Sep 22;30(18):3839. doi: 10.3390/molecules30183839 (PMC12472366; doi:10.3390/molecules30183839)
Supplement: Supplementary file 1 [file molecules-30-03839-s001.zip › molecules-3868811-supplementary.pdf]

Anti-Atrophic Effects of Dichotomine B from *Stellaria dichotoma* During Starvation-Induced Skeletal Muscle Atrophy

Figure S1. Solvent fractionation of *S. dichotoma* extract and HPLC analysis.

Figure S2. Isolation procedures using Diaion HP-20 chromatography.

Figure S3. HPLC chromatograms of *n*-butanol extract and isolated compounds **1**–**5**.

Figure S4. ^{1}H and ^{13}C NMR spectra of glucodichotomine B (**1**).

Figure S5. ^{1}H and ^{13}C NMR spectra of dichotomine B (**2**).

Figure S6. ^{1}H and ^{13}C NMR spectra of shaftoside (**3**).

Figure S7. ^{1}H and ^{13}C NMR spectra of dichotomine A (**4**).

Figure S8. ^{1}H and ^{13}C NMR spectra of isoshaftoside (**5**).

Figure S9. Cell viability of compounds **1**–**5** (30 μM) in C2C12 myotubes after incubation for 24 h.

Figure S10. Optimization of extraction solvents with various ethanol–water compositions for anti-atrophic activity during DEX-induced C2C12 myotube atrophy.

Figure S11. Cell viability of dichotomine B (**2**) in C2C12 myotubes after incubation for 24 h.

Table S1. Regression equations for isolated compounds **1**–**5**.

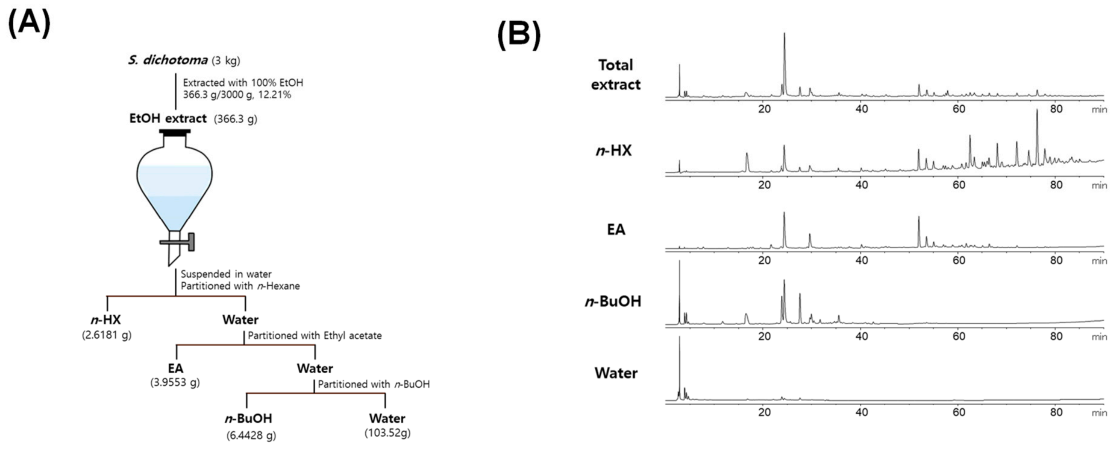

Figure S1. Solvent fractionation of *S. dichotoma* extract and HPLC analysis. (A) Solvent fractionation scheme and (B) HPLC chromatogram of each extract. *n*-HX: *n*-hexane extract, EA: ethyl acetate extract, *n*-BuOH: *n*-butanol extract, Water: water extract. HPLC conditions: Shiseido Capcell Pak UG120 C18 (250 × 4.6 mm); sample concentration: 10 mg/mL; mobile phase: A—acetonitrile + 0.1% TFA, B—water + 0.1% TFA; mobile phase conditions: 0-5 min, 5% A; 3-30 min, 5-30% A; 30-85 min, 30-95% A; 95-100 min, 95-100% A; flow rate: 1 mL/min; wavelength: 265 nm.

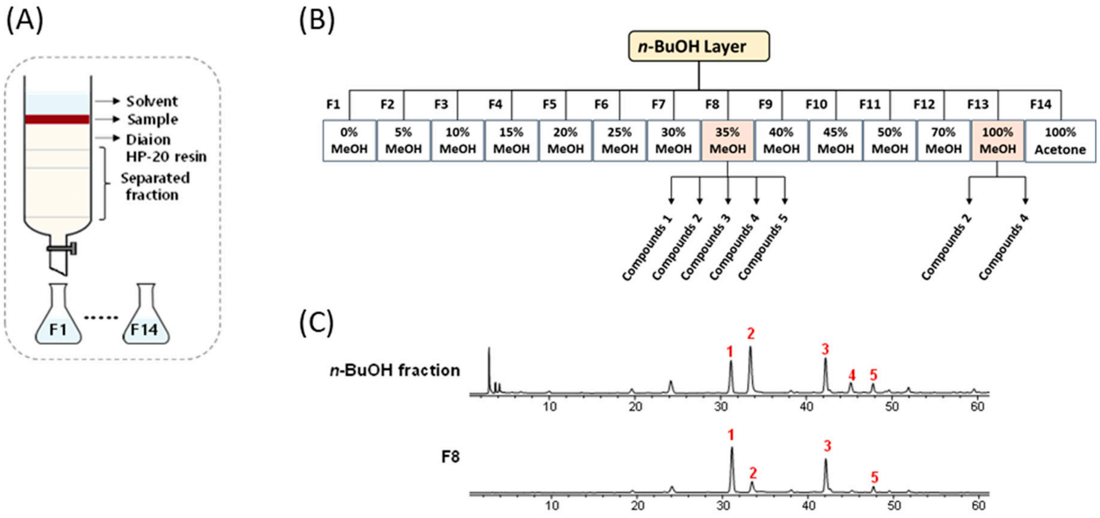

Figure S2. Isolation procedures using Diaion HP-20 chromatography. (A) Figure of Diaion HP-20 chromatography, (B) solvent elution of Diaion HP-20 chromatography, and (C) HPLC chromatograms of *n*-butanol extract and obtained sub-fractions F8 and F13 using Diaion HP-20 chromatography. HPLC conditions: Shiseido Capcell Pak UG C18 (250 × 4.6 mm); sample concentration: 5 mg/mL; mobile phase: A—acetonitrile + 0.1% TFA, B—water + 0.1% TFA; mobile phase conditions: 0-5 min, 5% A; 5-60 min, 5-20% A; 60-70 min, 20-95% A; flow rate: 1 mL/min; wavelength: 265 nm.

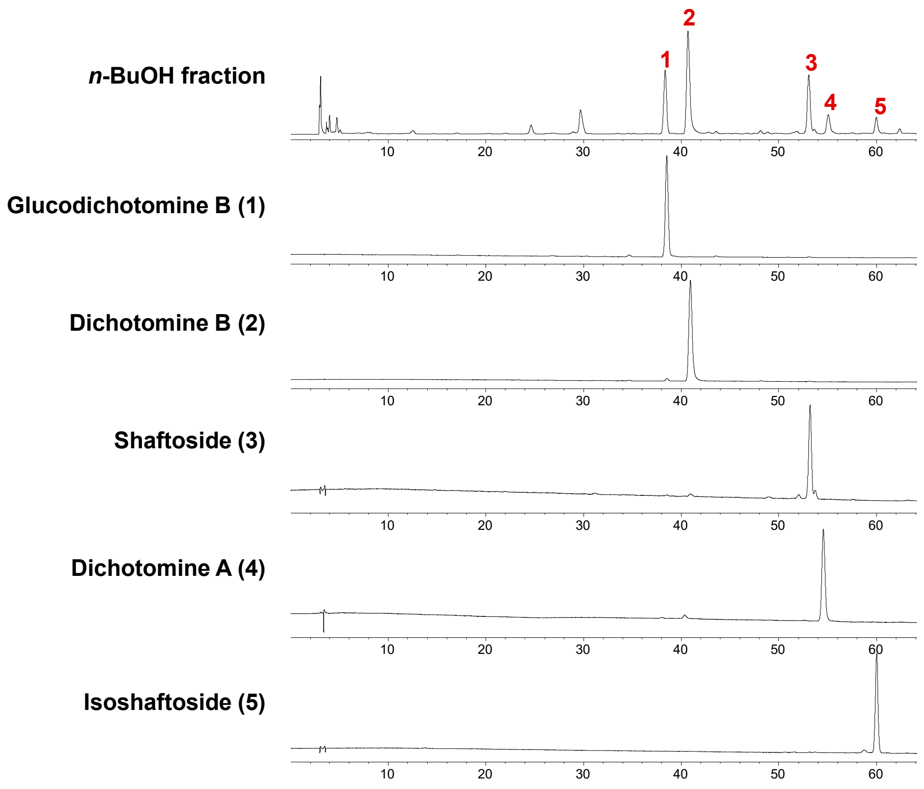

Figure S3. HPLC chromatograms of *n*-butanol extract and isolated compounds **1** - **5**.

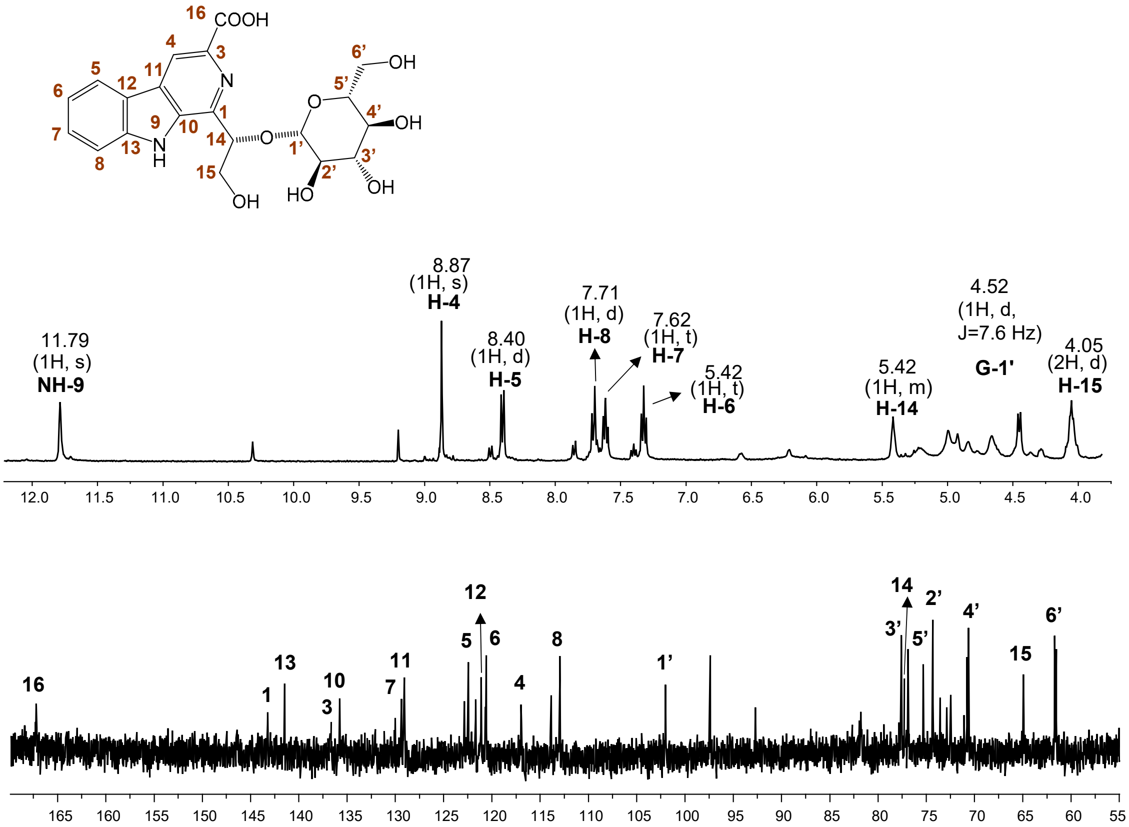

Figure S4. ^{1}H and ^{13}C NMR spectra of glucodichotomine B (**1**).

^{1}H NMR (400 MHz, DMSO-*d*$_6$): δ ppm 4.05 (2H, d, *J*= 7.9 Hz, H-15), 4.52 (1H, d, *J*=7.6 Hz, G-1'), 5.42 (1H, m, H-14), 7.42 (1H, t, H-6), 7.62 (1H, t, H-7), 7.71 (1H, d, *J*=7.4 Hz, H-8), 8.40 (1H, d, *J*=7.4 Hz, H-5), 8.87 (1H, s, H-4), 11.79 (1H, s, NH-9).

^{13}C NMR (100 MHz, DMSO-*d*$_6$): δ ppm 61.71 (G-6'), 64.95 (C-15), 70.63 (G-4'), 74.32 (G-2'), 76.88 (G-5'), 77.24 (C-14), 77.59 (G-3'), 102.00 (G-1'), 112.95 (C-8), 116.96 (C-4), 120.59 (C-6), 121.10 (C-12), 122.43 (C-5), 129.06 (C-11), 129.36 (C-7), 135.74 (C-10), 136.74 (C-3), 141.45 (C-13), 143.21 (C-1), 167.79 (C-16).

The structure of compound **1** was identified as glucodichtomine B (**1**) by comparing the NMR and MS spectral data with the literature [S1].

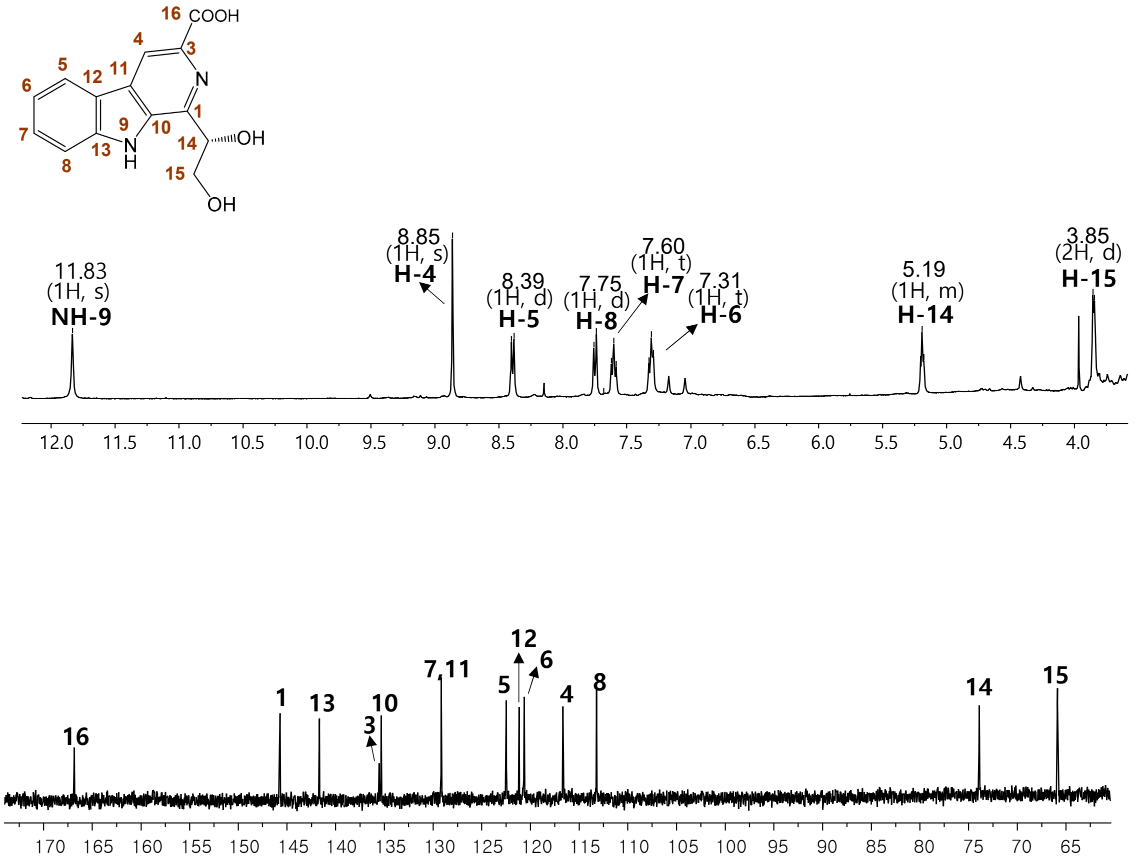

Figure S5. ^{1}H and ^{13}C NMR spectra of dichotomine B (**2**).

^{1}H NMR (400 MHz, DMSO-d_6): δ ppm 3.85 (2H, d, *J*=7.9 Hz, H-15), 5.19 (1H, m, H-14), 7.31 (1H, t, H-6), 7.60 (1H, t, H-7), 7.75 (1H, d, *J*=7.5 Hz, H-8), 8.39 (1H, d, *J*=7.5 Hz, H-5), 8.85 (1H, s, H-4), 11.83 (1H, s, NH-9).

^{13}C NMR (100 MHz, DMSO-d_6): δ ppm 66.85 (C-15), 73.90 (C-14), 113.19 (C-8), 116.63 (C-4), 120.62 (C-6), 121.15 (C-12), 122.46 (C-5), 129.13 (C-11), 129.13 (C-7) 135.31 (C-10), 135.51 (C-3), 141.66 (C-13), 145.69 (C-1), 166.82 (C-16).

The structure of compound **2** was identified as dichtomine B (**1**) by comparing the NMR and MS spectral data with the literature [S1].

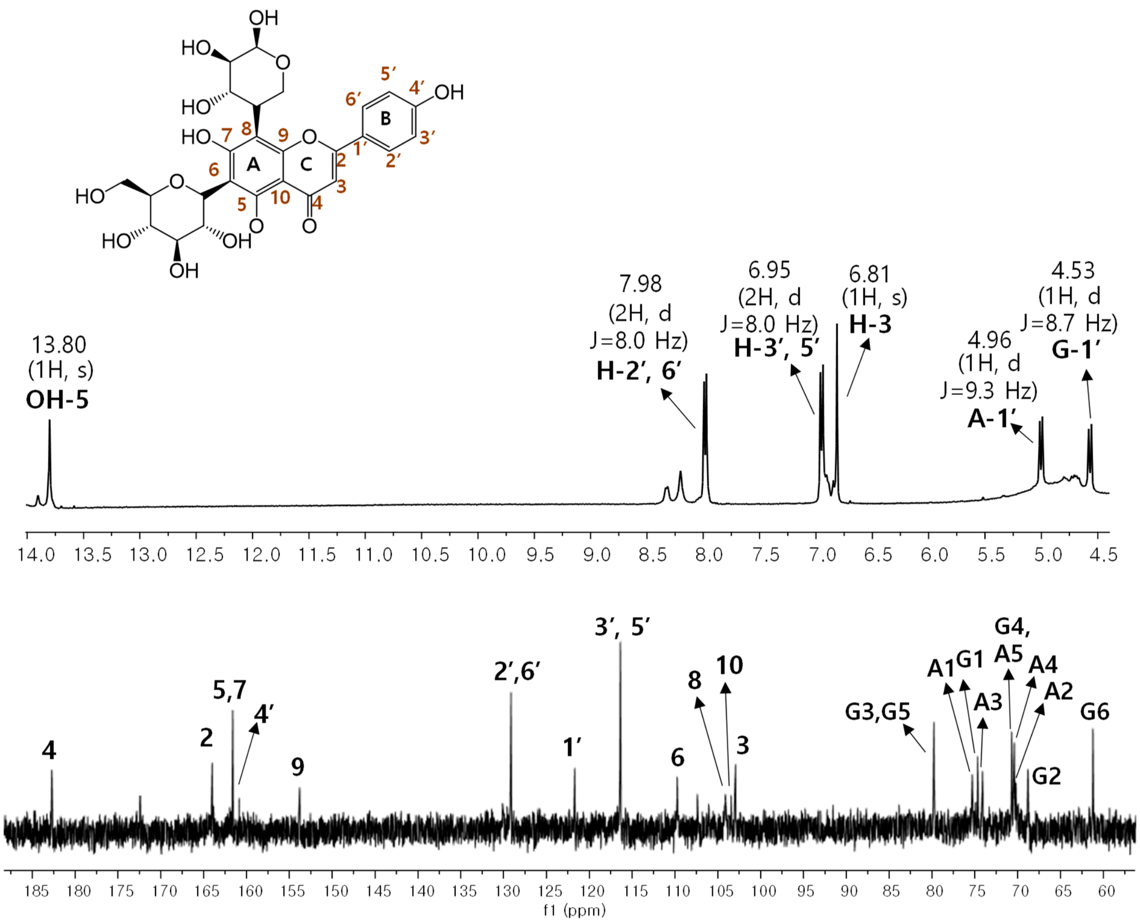

Figure S6. ^{1}H and ^{13}C NMR spectra of shaftoside (**3**).

^{1}H NMR (400 MHz, DMSO-*d₆*): δ ppm 4.53 (1H, d, *J*=8.7 Hz, G-1'), 4.96 (1H, d, *J*=9.3 Hz, A-1'), 6.81 (1H, s, H-3), 6.95 (2H, d, *J*=8.0 Hz, H-3', 5'), 7.98 (2H, d, *J*=8.0 Hz, H-2', 6'), 13.80 (1H, s, OH-5).

^{13}C NMR (100 MHz, DMSO-d_6): δ ppm 61.32 (G-2), 66.92 (G-6), 70.31 (A-2), 70.49 (A-4), 70.79 (A-5, G-4), 74.22 (A-3), 74.77 (G-1), 75.42 (A-1), 79.88 (G-3, G-5), 108.08 (C-3), 108.54 (C-10), 104.20 (C-8), 109.83 (C-6), 116.47 (C-3', 5'), 121.80 (C-1'), 129.24 (C-2',6'), 153.89 (C-9), 161.70 (C-4'), 164.11 (C-5, C-7), 182.88 (C-4).

The structure of compound **3** was identified as shaftoside (**3**) by comparing the NMR and MS spectral data with the literature [S2].

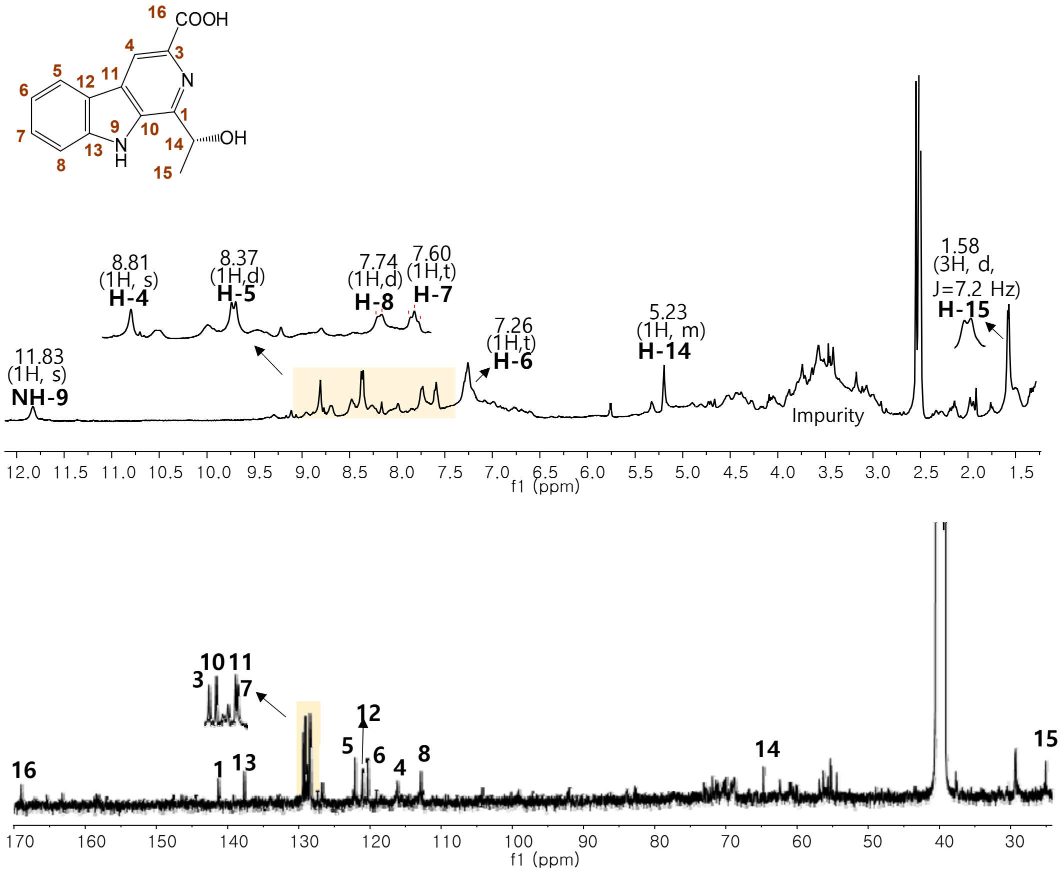

Figure S7. ^{1}H and ^{13}C NMR spectra of dichotomine A (**4**).

^{1}H NMR (400 MHz, DMSO-d_6): δ ppm 1.58 (3H, d, *J*=7.2 Hz, H-15), 5.23 (1H, m, H-14), 7.26 (1H, t, H-6), 7.60 (1H, t, H-7), 7.74 (1H, d, *J*=7.0 Hz, H-8), 8.37 (1H, d, *J*=7.0 Hz, H-5), 8.81 (1H, s, H-4), 11.83 (1H, s, NH-9).

^{13}C NMR (100 MHz, DMSO-d_6): δ ppm 25.04 (C-15), 64.80 (C-14), 113.14 (C-8), 116.27 (C-4), 120.52 (C-6), 121.29 (C-12), 122.34 (C-5), 128.63 (C-7), 128.70 (C-11), 129.37 (C-10), 129.61 (C-3), 137.91 (C-13), 141.56 (C-1), 169.20 (C-16).

The structure of compound **4** was identified as dichtomine A (4) by comparing the NMR and MS spectral data with the literature [S1].

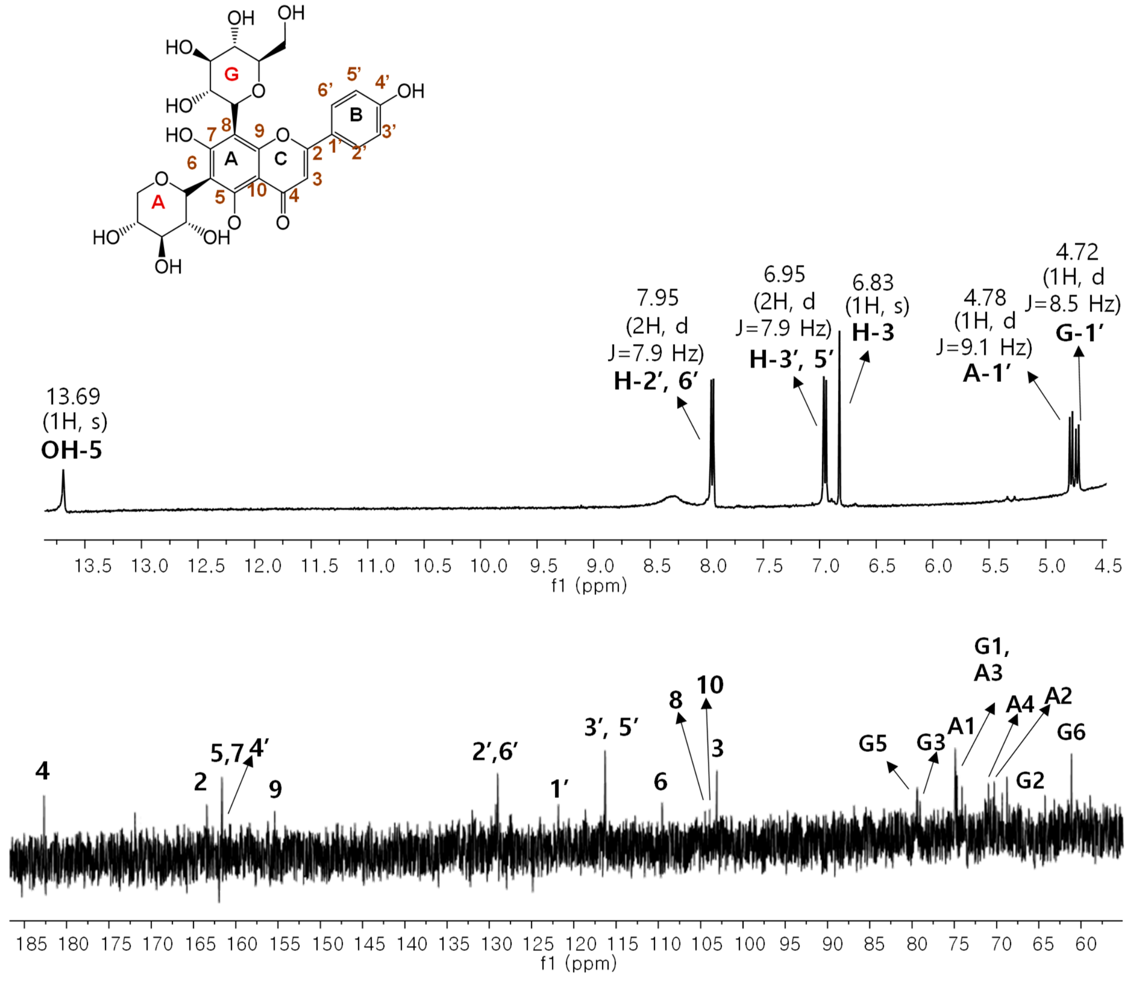

Figure S8. ^{1}H and ^{13}C NMR spectra of isoshaftoside (**5**).

^{1}H NMR (400 MHz, DMSO-*d*$_6$): δ ppm 4.72 (1H, d, *J*=8.5 Hz, G-1'), 4.78 (1H, d, *J*=9.1 Hz, A-1'), 6.83 (1H, s, H-3), 6.95 (2H, d, *J*=7.9 Hz, H-3', 5'), 7.95 (2H, d, *J*=7.9 Hz, H-2', 6'), 13.69 (1H, s, OH-5).

^{13}C NMR (100 MHz, DMSO-*d*$_6$): δ ppm 61.25 (G-6), 68.88 (G-2), 70.96 (A-2), 71.07 (A-4), 74.77 (A-3, G-1), 74.99 (A-1), 79.17 (G-3), 79.48 (G-5), 108.23 (C-3), 104.13 (C-10), 104.95 (C-8), 109.67 (C-6), 116.40 (C-3', 5'), 121.93 (C-1'), 129.11 (C-2', 6'), 156.28 (C-9), 160.84 (C-4), 161.71 (C-5, C-7), 163.51 (C-2), 182.76 (C-4).

The structure of compound **5** was identified as isoshaftoside (**5**) by comparing the NMR and MS spectral data with the literature [S2].

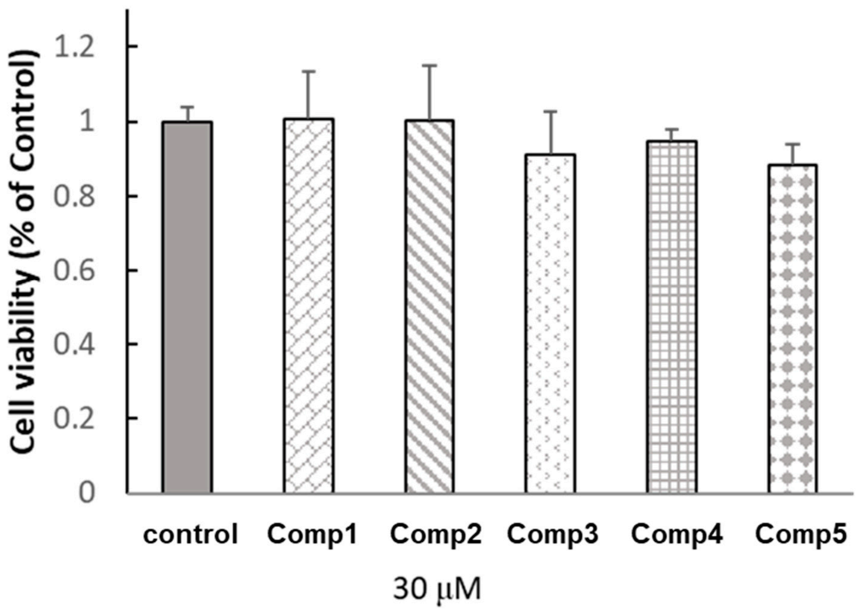

Figure S9. Cell viability of compounds **1**–**5** (30 μM) in C2C12 myotubes after incubation for 24 h.

Extraction for Optimization of Ethanol/Water Solvent Ratios

Ten grams of *S. dichotoma* powder was extracted for 3 h under reflux with 100 mL of the following solvents: 100% water, 25% aqueous ethanol, 50% aqueous ethanol, 75% aqueous ethanol, and 100% ethanol. The extract was filtered, dried under a rotary evaporator, and freeze-dried.

HPLC Analysis

Each extract was dissolved at a concentration of 100 mg/mL in dimethyl solfoxide (DMSO), then diluted tenfold with methanol to a concentration of 10 mg/mL. Isolated compounds **1**–**5** were used to prepare standard solutions. Each compound was dissolved in methanol at a concentration of 1 mg/mL and serially diluted, as shown in Supplementary Table S1. Linear regression equations were calculated using y = ax ± b, where x is the concentration and y is the peak area of each compound. Linearity was established by the coefficient of the equation (R^2).

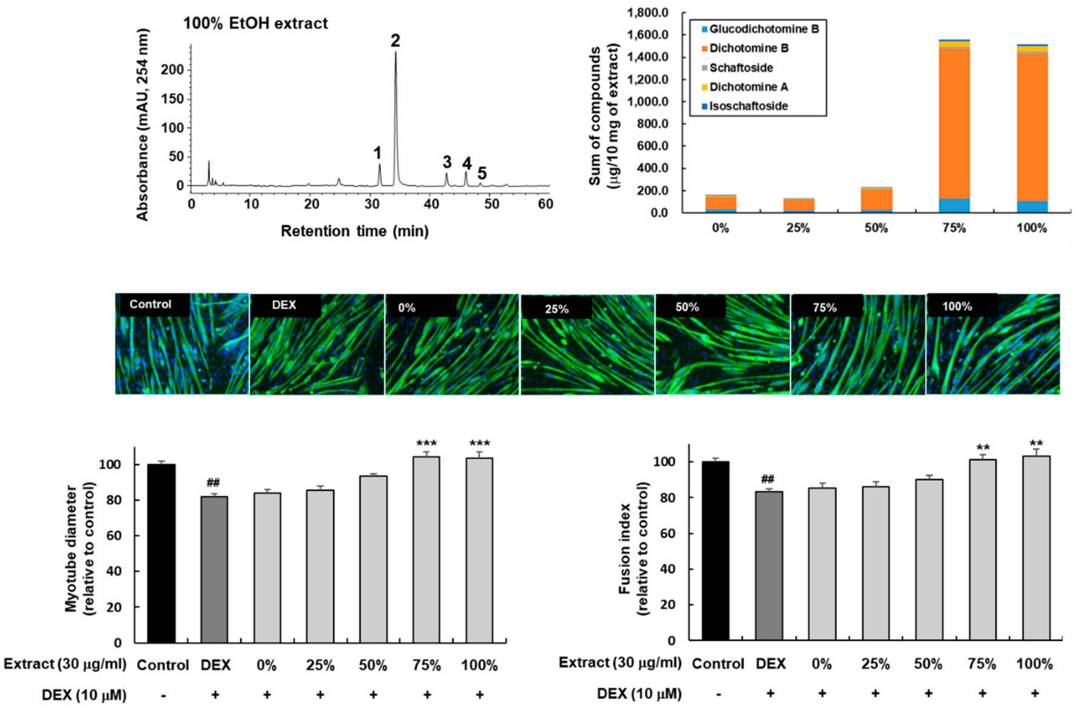

Figure S10. Optimization of extraction solvents with various ethanol–water compositions for anti-atrophic activity during DEX-induced C2C12 myotube atrophy. **(A)** An HPLC chromatogram of the 100% ethanol extract. HPLC analysis was performed using an Agilent Technologies 1200 system equipped with an automatic injector, a column oven, and a DAD detector. A Shiseido Capcell Pak UG120 C18 column (250 × 4.6 mm, 5 μm, Shiseido, Tokyo, Japan) was used. The temperature was maintained at 40 °C, with an injection volume of 20 μL and a flow rate of 1 mL/min. The mobile phase was composed of acetonitrile containing 0.1% trifluoroacetic acid (A) and water containing 0.1% trifluoroacetic acid (B). The gradient elution conditions were as follows: 0 min A:B—5:95, v/v; 5 min A:B—5:95; and 60 min A:B—20:80. The detection wavelength was 265 nm. **(B)** The sums of compounds **1**–**5** in extracts with various extraction conditions. **(C)** Immunofluorescence staining of MHC in C2C12 myotubes after cotreatment with DEX and each extract (30 μg/mL) for 24 h. Cell morphology and relative changes in **(D)** the myotube diameter and **(E)** fusion index in dexamethasone-induced C2C12 myotubes cotreated with each extract. These results are presented

as means ± SDs (n = 3). ## p < 0.01 vs. control. ** p < 0.01 vs. dexamethasone treatment. *** < 0.001 vs. dexamethasone treatment.

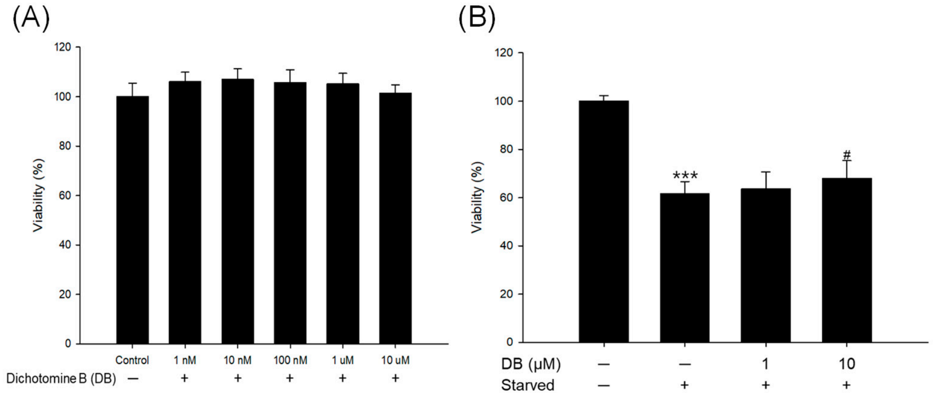

Figure S11. Cell viability of dichotomine B (**2**) in C2C12 myotubes after incubation for 24 h. (A) Cell viability of dichotomine B (1 nM–10 μM) and (B) cell viability in starved conditions (1 or 10 μM).
Table S1

Table S1. Regression equations for isolated compounds **1** - **5**.

Compound	Regression equation	R^2	Linear range (μg/mL)
Glucodichotomine B (**1**)	y = 6.7860x + 5.1833	0.9998	7.8125 – 125
Dichotomine B (**2**)	y = 3.9332x + 350.54	0.9999	62.5 – 2000
Shaftoside (**3**)	y = 19.93x + 4.675	0.9999	1.953125 – 31.25
Dichotomine A (**4**)	y = 11.623x + 6.1167	0.9997	3.90625 – 62.5
Isoshaftoside (**5**)	y = 7.7295x + 3.0208	0.9992	1.953125 – 31.25

References

1S. Morikawa, T.; Sun, B.; Matsuda, H.; Wu, L. J.; Harima, S.; Yoshikawa, M. Bioactive constituents from Chinese natural medicines. XIV. New glycosides of β-carboline-type alkaloid, neolignan, and phenylpropanoid from Stellaria dichotoma L. var. lanceolata and their antiallergic activities. *Chem. Pharm. Bull.* **2004**, *52*, 1194-9. doi:10.1248/cpb.52.1194.

2S. Simirgiotis, M. J.; Schmeda-Hirschmann, G.; Bórquez, J.; Kennelly, E. J. The Passiflora tripartita (Banana Passion) fruit: a source of bioactive flavonoid C-glycosides isolated by HSCCC and characterized by HPLC–DAD–ESI/MS/MS. *Molecules* **2013**, *18*, 1672-1692. doi:10.3390/molecules18021672.
